# Patients’ Need for Tailored Comparative Health Care Information: A Qualitative Study on Choosing a Hospital

**DOI:** 10.2196/jmir.4436

**Published:** 2016-11-28

**Authors:** Nicolien C Zwijnenberg, Michelle Hendriks, Evelien Bloemendal, Olga C Damman, Judith D de Jong, Diana MJ Delnoij, Jany JD Rademakers

**Affiliations:** ^1^Netherlands Institute for Health Services Research (NIVEL)UtrechtNetherlands; ^2^Department of Public and Occupational Health and EMGO+ Institute for Health and Care ResearchVU University Medical CenterAmsterdamNetherlands; ^3^Quality InstituteNational Health Care InstituteDiemenNetherlands; ^4^Tilburg School of Social and Behavioral SciencesTranzoTilburg UniversityTilburgNetherlands; ^5^School for Public Health and Primary Care (CAPHRI)Department of Family MedicineMaastricht UniversityMaastrichtNetherlands

**Keywords:** patients, decision making, choice behavior, qualitative research, quality of health care, hospitals

## Abstract

**Background:**

The Internet is increasingly being used to provide patients with information about the quality of care of different health care providers. Although online comparative health care information is widely available internationally, and patients have been shown to be interested in this information, its effect on patients’ decision making is still limited.

**Objective:**

This study aimed to explore patients’ preferences regarding information presentation and their values concerning tailored comparative health care information. Meeting patients’ information presentation needs might increase the perceived relevance and use of the information.

**Methods:**

A total of 38 people participated in 4 focus groups. Comparative health care information about hip and knee replacement surgery was used as a case example. One part of the interview focused on patients’ information presentation preferences, whereas the other part focused on patients’ values of tailored information (ie, showing reviews of patients with comparable demographics). The qualitative data were transcribed verbatim and analyzed using the constant comparative method.

**Results:**

The following themes were deduced from the transcripts: number of health care providers to be presented, order in which providers are presented, relevancy of tailoring patient reviews, and concerns about tailoring. Participants’ preferences differed concerning how many and in which order health care providers must be presented. Most participants had no interest in patient reviews that were shown for specific subgroups based on age, gender, or ethnicity. Concerns of tailoring were related to the representativeness of results and the complexity of information. A need for information about the medical specialist when choosing a hospital was stressed by several participants.

**Conclusions:**

The preferences for how comparative health care information should be presented differ between people. “Information on demand” and information about the medical specialist might be promising ways to increase the relevancy and use of online comparative health care information. Future research should focus on how different groups of people use comparative health care information for different health care choices in real life.

## Introduction

The Internet is increasingly being used to provide patients with information about the quality of care of different health care providers [1]. The main philosophy behind this quality information—also known as comparative health care information—is that it enables patients to make well-informed health care choices. In health care systems where patients have the right to choose their own providers, quality information can support patients in selecting the best providers and patients can thereby stimulate health care quality improvement [2]. In addition, the information in itself is also thought to empower patients in becoming autonomous health care consumers [3].

Although comparative health care information is widely available internationally [4-7], and patients have been shown to be interested in this information [6], its effect on patients’ decision making is still limited [6-10]. A systematic review by Faber and colleagues [6] showed that quality information influenced patients’ health care provider choice in less than 5% of cases. Reasons why patients have been reluctant to embrace comparative health care information include unawareness of the availability of information, problems with timely access of the information, difficulty in understanding the complex information, and perceiving it as irrelevant [6,9]. Involving patients in developing comparative health care information is important in order to meet patients’ information needs. A body of research has focused on patients’ preferences for the content of quality information [11-16]. These studies revealed, for example, that patients value information on both technical and interpersonal quality [15], and that the importance attached to choice aspects differs between patients [14].

However, how information is presented can be as influential as what information is presented when making health care choices [17]. Hibbard and Peters [18(p414)] stated that “the challenge is not merely to communicate accurate information, but to understand how to present and target that information so that it is actually used in decision making.” In their conceptual model, they described 3 process goals to enhance the use of comparative health care information: lowering the cognitive effort needed to process the information, making clear what a choice means for people in real life, and making information more salient by highlighting its meaning.

These 3 goals can be accomplished through several presentation strategies, of which we will address a few [18]. The cognitive effort can by reduced by providing a limited amount of information [17] and by using data displays that are easy to evaluate [18]. It has been shown that humans can process and use only a limited amount of information—approximately 4 to 6 aspects—when making choices [11,18,19]. Using displays that transform the information into an evaluative good/bad scale might help people in processing and understanding the information, such as ordering health care providers by performance from best to worse. However, patients’ preferences concerning the number of, and order in which, health care providers have to be displayed on websites remain unclear.

Tailoring comparative health care information might contribute to all 3 goals [18]. Kreuter and Skinner [20(p1)] defined tailoring as “any combination of information or change strategies intended to reach one specific person, based on characteristics that are unique to that person, related to the outcome of interest” (p 1). Literature supports the effectiveness of tailored health information or interventions, for example, in the context of tailored communication for cancer patients or tailored interventions to promote health behavior [21-24]. For comparative health care information, tailoring could imply that patients are shown quality information about health care delivered to patients with comparable demographic characteristics. For example, only information that is about patients of comparable age or same ethnicity could be shown. Although the merits of tailoring information might be evident in some specific health care contexts, it is unclear how patients value tailored comparative health care information.

Our study aimed to explore patients’ information presentation preferences as well as their values regarding tailored comparative health care information. Information about choosing a hospital for an elective surgery (ie, a total hip surgery) was used in this study as a case example. In the case of an elective surgery, people often have sufficient time to search for information and are able to make well-informed choices. The following research questions were addressed:

1. What are patients’ preferences concerning the presentation of comparative health care information. More specifically, what are their preferences for the number of, and the order in which, health care providers are presented on websites showing comparative health care information?

2. What are patients’ values regarding tailoring, such as presenting patient reviews of patient subgroups with comparable demographic characteristics (age, gender, or ethnicity)?

## Methods

### Design

This study was part of a larger research project in which we collaborated with the Dutch Federation of Patients and Patient Organizations (Nederlandse Patiënten en Consumenten Federatie; NPCF) in optimizing their website Consumentendezorg.nl. More specifically, the project focused on comparative health care information on total hip, knee, or cataract surgery. To answer the research questions, we performed focus groups with patients who underwent hip, knee, or cataract surgery and with members of an access panel of Netherlands Institute for Health Services Research (Nederlands instituut voor onderzoek van de gezondheidszorg; NIVEL). The focus groups took place in March 2010 at NIVEL. Each session lasted approximately 2 hours, was facilitated by the same team of investigators (EB moderator; NZ secretary), and followed a structured interview protocol. Participants received a €15 gift voucher and a summary of the main findings.

### Recruitment of Participants

Participants were recruited in 2 ways. First, as part of the larger research project, we posted calls on websites of patient organizations for orthopedic patients and patients with eye disorders, on websites of Dutch associations for senior citizens, and on the website of the NPCF. Respondents to a questionnaire that was part of the research larger project could also enroll themselves in this study by reporting their interest at the end of the questionnaire [13]. Via this route, 56 patients were included.

We anticipated that 56 potential participants would not be enough to reach saturation; therefore, we also invited 139 members of the NIVEL “Insurants Panel” by mail. The Insurants Panel is an access panel installed and managed by NIVEL and consists of a cohort of insurants from one of the biggest Dutch health insurers. The aim of the panel is to gather information on patients’ experiences with, and expectations of, health care in general and their health insurer in particular. Members were recruited for the panel through an announcement in the magazine of the health insurer and by calling them and asking them to join the panel. Compliance with privacy regulations was approved by the Dutch Data Protection Authority (nr. 1309664). For this study, we selected 139 members who were 40 years or older and who had a travel time of less than 45 minutes to the interview location. We used this age criterion because this group would most likely have experience with choosing a hospital. Also, the case example of choosing a hospital for total hip surgery is less relevant for younger people.

Participation in the focus groups took place on a voluntary basis and informed consent of the participants was obtained. Ethical approval of the study was not required because research using interviews that are not taxing or hazardous for participants (ie, the once-only answering of questions that do not constitute a serious encroachment on the participant) is not subject to the Dutch Medical Research Involving Human Subjects Act (Wet medisch-wetenschappelijk onderzoek met mensen).

### Interview Guide

The focus group discussion was divided into 2 rounds addressing the different research questions (see Textbox 1). The first part of the interview focused on participants’ preferences for information presentation, whereas the second part focused on participants’ values regarding presenting comparative health care information about patients with comparable demographic characteristics (ie, tailoring). The website of the NPCF was used as an illustration during the second part of the meeting. The example provided comparative health care information for total hip and knee surgery. The information consisted of orthopedic patients’ experiences with the conduct of medical specialists (ie, orthopedists), conduct of the nurses, and information on medicines. Distance to the hospital, the number of hip replacements per year, and the number of knee replacements per year were also displayed. We showed 2 Web pages: one displaying comparative health care information (in columns) for all hospitals within 50 km distance (in rows), and one displaying comparative health care information (in rows) for 3 hospitals (in columns).

**Interview protocol for focus groups.**

**General Introduction**

- Introduction of 2 researchers (moderator and secretary); background information about study; announcements

**Part 1**

Introduction Part 1

- Introducing oneself and previous experiences with choosing a hospital: “What is your experience with choosing a hospital?”
- Introducing test case (choosing a hospital for a total hip replacement surgery)
- “Imagine that you have to select a hospital for hip replacement surgery. Would you use a website, such as consumentendezorg.nl, that provides comparative information?”
- “Suppose you are using this website. Would you prefer to compare different hospitals and make a choice or would you prefer to see quality information about only one hospital?”
- “How many hospitals would you prefer to see quality information about?”
- “Would you like to see hospitals ranked in alphabetical order, on distance, from good to bad, or ranked according to another criterion?”

**Part 2 (the website consumentendezorg.nl of the NPCF was used as an illustration)**

Introduction Part 2

- “Suppose that, when you are choosing a hospital for a hip replacement surgery, you can fill in your age on the website. For example, 65 years or older. By doing this, you receive an overview of quality information of hospitals, based on reviews of patients of the same age. What is your opinion about this kind of information?”
- “Suppose that you can fill in information about your gender. By doing this, you receive quality information of hospitals based on reviews of people with the same gender. What is your opinion about this kind of information?”
- “Are there other subgroups of which you would like to see quality information of hospitals?” (When ethnicity, educational level and health status were not mentioned: “What do you think, for example, of quality information of hospitals based on reviews of people with a comparable (high or low) level of education, with the same ethnical background, or with a comparable (low or high) health status?”)

**Conclusion**

- After seeing more information about the website consumentendezorg.nl, would you make use of this website?
- Summary of the group discussion

### Analysis

Sessions were audiotaped and notes were taken with the participants’ consent. All audiotaped sessions were transcribed verbatim. The constant comparative method, one of the core analysis techniques in the grounded theory approach [25], was used to analyze the data. First, the transcripts of the focus groups were read and open-coded by 2 researchers independently (NZ and EB). The coded transcripts were compared and a code tree was created. Next, all focus groups were coded using this code tree by the same researchers. The codes were compared; where differences in themes occurred, consensus was reached through discussion with a third researcher (MH).

## Results

### Participants

A total of 38 people participated in 4 focus groups (see Table 1). Participants in focus groups 1 and 2 were patients that underwent or had to undergo total hip, total knee, or cataract surgery. Participants of focus groups 3 and 4 were members of the NIVEL Insurants Panel. In the fourth focus group, no new information was gathered (ie, data saturation was reached).

**Table 1:** Characteristics of participants.

Characteristic		Group 1 (n=7)	Group 2 (n=11)	Group 3 (n=9)	Group 4 (n=11)	Total (N=38)
**Gender, n (%)**						
	Men	3 (43)	5 (46)	6 (67)	7 (64)	21 (55)
	Women	4 (57)	6 (56)	3 (33)	4 (36)	17 (45)
Age (years), mean (SD)		64.0 (7.3)	66.1 (10.9)	70.1 (6.7)	64.5 (12.4)	66.2 (9.9)
**General health status, n (%)**						
	Excellent	0 (0)	1 (9)	0 (0)	1 (9)	2 (5)
	Very good	1 (14)	2 (18)	2 (22)	1 (9)	6 (16)
	Good	5 (71)	5 (46)	6 (67)	8 (73)	24 (63)
	Fair	1 (14)	3 (27)	1 (11)	1 (9)	6 (16)
	Poor	0 (0)	0 (0)	0 (0)	0 (0)	0 (0)
**Education,^a^ n (%)**						
	Low	0 (0)	0 (0)	0 (0)	1 (9)	1 (3)
	Average	3 (43)	4 (36)	3 (33)	3 (27)	13 (34)
	High	4 (57)	7 (64)	6 (67)	7 (64)	24 (63)
**Use of Internet, n (%)**						
	No use of Internet	1 (14)	0 (0)	0 (0)	1 (9)	2 (5)
	Monthly	0 (0)	0 (0)	0 (0)	0 (0)	0 (0)
	Weekly	0 (0)	2 (18)	1 (11)	0 (0)	3 (8)
	Daily	6 (86)	9 (82)	8 (89)	10 (91)	33 (87)

^a^ Low: primary school, lower level of secondary school; average: lower vocational training, intermediate vocational training or higher level of secondary school; high: higher vocational training or university.

The mean age of the 21 men and 17 women was 66.2 (SD 9.9) years, ranging from 46 to 95 years. The majority of the participants (63%, 24/38) graduated from higher vocational training or had an academic degree, which is not representative of the general population. The cohort was also fairly healthy; only 16% (6/38) perceived their general health status to be fair. Most participants used the Internet on a daily basis (87%, 33/38). Only 2 participants had never used the Internet. Of the participants in groups 1 and 2, 10 underwent total hip surgery, 7 had total knee surgery, and 4 had cataract surgery. Two participants were on a waiting list to undergo one of these surgeries. Of the members of the Insurants Panel, 12 participants underwent an elective surgery at least once in their life, of which 1 participant underwent total knee surgery and 1 had cataract surgery. Six participants had experience with choosing a hospital for consulting a medical specialist or outpatient treatments. Only 1 participant had no experience with choosing a hospital.

### Themes

The following main themes emerged from the analysis: (1) number of health care providers to be presented, (2) order in which health care providers are presented, (3) relevancy of tailoring patient reviews, and (4) concerns about tailoring. Although we presented the choice of a hospital for hip surgery as a case example, the discussion revolved around choosing a hospital in general. Therefore, we present the results for all participants together without distinguishing between participants based on the type of surgery they had or the method of recruitment.

#### Number of Health Care Providers

Most participants preferred a website with an overview of information about different health care providers, rather than information about one specific hospital only. Concerning the number of providers to be presented, different preferences emerged. Some wanted to compare approximately 5 providers, sometimes supplemented with a more specialized provider. Most of these participants wanted to select these providers based on the distance from their home to the hospital. On the other side, one participant preferred to compare all possible providers including providers from abroad. Others preferred to decide for themselves how many providers are shown:

So I think about five or six. That I think will be sufficient. At a certain point it will become a little too much.

female, group 2

This website is limited to hospitals in the Netherlands. There are also <hospitals> in Germany, especially in a number of cities. A link to their websites would be useful if they exist.

male, group 1

Let me choose a number of hospitals, from a very long list, which I want to include in my comparison.

male, group 2

The complexity of the disease/surgery, the clarity of the overview, and the results of the search could influence the preferred number of providers to be presented:

If I would need a less standard surgery, I would search harder than when I have the feeling: a hip is a pretty routine surgery.

female, group 2

I would compare five hospitals and if the results offer little choice, expand it to 10 or more.

male, group 3

#### Order of Health Care Providers

As for the order of health care providers, participants also varied in their preferences. Some liked to see providers ordered from short to long distance, a few preferred to see providers ordered from good to bad on a specific quality criterion, whereas others wanted to decide themselves how providers were ordered:

I would prefer distance.

female, group 3

Hospitals ordered from 10 to 0...so from good to bad.

male, group 4

I think there should be a choice in order, you must be able to decide if you prefer an alphabetical order, or geographical, or ordered on quality of this or that.

male, group 4

#### Relevancy of Tailoring Information

We asked participants what they thought about only presenting patient reviews that were given by patients of comparable age, gender, or ethnicity. Three different opinions were identified. The majority did not prefer subgroup-specific presentation of patient reviews for any of these characteristics:

Why would the opinion of a seventy-year-old patient be more important to me compared to an opinion of a 40-year-old? There are so many essential factors.

male, group 1

Do I find it important that a 60-year-old patient with a Turkish background is satisfied about their hip replacement or a 50-year-old Dutchman, I don’t think it matters.

male, group 1

Some welcomed subgroup-specific information in all cases and a minority would prefer subgroup-specific information only in the case of a significant effect:

The chance of complications increases when you are older; more chance of infections and this [older] patient will probably give a review on how the hospital dealt with this.

male, group 2

If there is a significant effect, it could be interesting to show it. But if research revealed that there is no significant effect, there is no need to present it.

female, group 2

Other participants were interested in information differentiation if differences were related to physical differences or the reason for the surgery:

[Regarding differentiation on gender] If it is anatomically a different kind of hip, but I think it is the same.

female, group 1

I would be more interested in whether someone had a hip replacement after a trauma or whether surgery was performed because of a degenerative process.

female, group 2

#### Concerns of Tailoring

Several participants were concerned about the representativeness of information when only subgroup-specific information would be provided. These concerns were mainly related to the smaller sample sizes that result from tailoring, and a few participants felt that the results could be biased:

The numbers will decrease. If you split this information, what is then the value?

male, group 1

I don’t see the relevance of age. You only get a more limited answer.

male, group 3

For some participants, it would be too complicated to present subgroup-specific information on websites:

For goodness’ sake, keep the website as simple as possible.

male, group 3

#### Level of Information

It is important to note that the need to compare medical specialists instead of hospitals was a recurring topic discussed in all 4 groups. Most participants wanted to choose a particular specialist instead of a hospital:

The problem with this information is that there’s a lot of information on results of hospitals and specialties overall, but there’s no information about specialist A or specialist B.

male group 1

The specialist did not form the basis for all participants, however, as one participant made clear:

No, it’s not about the specialist. It’s about the hospital. There you will find a certain specialism. It depends on your abilities, the distance, your physical condition, the type of disease you have. So of course you will look at hospitals.

male, group 4

## Discussion

### Principal Results

Our qualitative study focused on patients’ information presentation preferences and values regarding tailored comparative health care information. Comparative health care information about total hip surgery in hospitals was used as a case example. Participants’ preferences differed concerning how many and in which order health care providers should be presented on a website. Most participants had no interest in tailored information based on age, gender, or ethnicity. The need for more information about the medical specialist when choosing a hospital was stressed by several participants.

### Comparison With Prior Work

Previous studies have shown that the order of information presentation can greatly influence the attention people pay to particular parts of that information [26]. As for the effect of ordering health care providers in comparative health care information, research of Damman and colleagues [27] showed that ordering providers alphabetically resulted in more effective use of the information (eg, respondents chose the top-performing provider more often) than ordering on performance. However, other studies showed positive effects of ordering providers on performance [28,29]. These differences might reflect, as we found, that people differ in their preferences concerning the order in which health care providers should be presented on websites.

Although tailoring might enhance the relevance of online comparative health care information, our findings showed that the majority of participants had no interest in tailored information. That is, they did not value reviews of patients of the same age, gender, or ethnicity. These results are not in line with our expectations. Earlier studies about health care choices showed that patients, in general, prefer information about people comparable to themselves in terms of age, socioeconomic status, or geographic area [11]. For now, we can only speculate about these contradictory findings. Perhaps patients do not see the added value of tailored comparative health care information because the disadvantages (eg, more complicated information and less patient reviews available) outweigh the advantages (eg, more personally relevant information). Some participants were interested in information provided by patients with comparable preconditions before surgery. Maybe they felt that disease characteristics are more related to the reported quality indicators than demographics. As tailoring entails presenting information according to characteristics that are related to the outcome of interest [20], it should be determined in future research whether tailoring comparative health care information based on disease characteristics (eg, health status before surgery) is perceived as more relevant.

One comment persistently made by several participants was that they wanted information about the medical specialist rather than the hospital. The importance of the medical specialist in health care decision making [13,30] and the need for information about specialists’ interpersonal and communication skills and expertise [14] is also revealed in other research. The availability of online doctor-rating websites is growing and these websites have gained popularity among patients internationally [31-34]. Although “doctor bashing” is a concern regarding these websites, most studies show that these websites provide favorable ratings of doctors [31,32] and evidence exists that these ratings correlate with survey measures of patient experiences [35,36]. Other drawbacks of these rating websites are that reviews of doctors are often based on only a few reviews [37], and privacy issues of individual doctors are at stake. Drawbacks of public disclosure of success rates of medical specialists also exist, for example, motivating surgeons to avoid high-risk patients and unjustly damaging specialist’s reputations. Future research should examine the effects of presenting comparative health care information for individual health care providers instead of hospitals on the use of the information by patients. Also, the entitlement of patients to access this relevant information should be carefully balanced against potential side effects.

This study focused on choosing a hospital in the case of an elective surgery that was not life threatening. It could be that preferences for information presentation are different when people have to choose a health care provider in a more acute and/or life-threatening situation. In acute situations, people have fewer or no opportunities to find and process comparative health care information. This stresses the importance of making information as easy to understand as possible. It has also been shown that people have different information needs depending on the disease or condition [13,38]. Whether preferences for how information should be presented also differ among diseases is yet unknown.

### Implications for Website Designers

Our results have implications for website designers. First, we recommend involving the intended users in the development of comparative health care information. Second, it is important to limit the amount of information that is presented. Finally, participants expressed different information presentation preferences, information needs, and values regarding tailored comparative health care information. This emphasizes the need for flexible, user-friendly websites, or “information on demand.” A review by Vaiana and McGlynn [39] also mentioned that websites need to be responsive to different users and that the “one-size-fits-all” approach needs to be challenged. By providing information on demand, patients themselves can have an active role in the health care information that is supplied [39]. Patients can select, for example, how many health care providers are shown, how providers are ordered, or which quality aspects are shown. Seeing tailoring in a broader perspective rather than in the classical definition in which information is tailored to someone’s unique characteristics, providing information on demand, or tailoring information presentation might help to meet patients’ information needs and increase the relevancy of online comparative health care information.

### Limitations

The strength of our study lies in the high number of participants in our qualitative research and the richness of opinions expressed by these participants. We used the interactional nature of focus groups to unravel participants’ opinions [40]. A limitation of our study is that participants were not representative of the general population. Participants were highly educated, which might be reflected in the expressed concerns about representativeness of data and the preference for tailored information only in the case of significant differences between subgroups. These 2 constructs (ie, representativeness and significant differences) might not come so easily to mind of people with a low education. Although we did not ask for their ethnic background, most participants appeared to be of Dutch origin. We do not know whether the preferences of ethnic minorities are different, especially when it comes to tailoring information based on ethnicity. Second, we only analyzed participants’ perceptions about online comparative health care information. It is well known that a person’s perception may not align with his/her actual behavior in practice. Although almost all participants had experience with choosing a hospital, most participants were not facing an actual hospital choice for hip surgery at the time of the focus groups. They either already had undergone this surgery or had no experience with hip problems. From the decision-making literature, it is known that people often have difficulties anticipating their preferences should their needs change [41]. The theory of constructed preference posits that preferences are often constructed in the process of deciding [42]. Third, preferences of people appear to be sensitive to the way a choice is described or what information is provided [18]. This means that the content and presentation format of the NPCF website might have influenced the thoughts and ideas of participants in some way. These limitations have to be taken into account when interpreting our results. It also stresses the need for research that investigates in real life how people use comparative health care information in health care choices.

### Conclusions

The preferences for how comparative health care information should be presented differ between people. This is true for how many and in which order health care providers should be presented and whether the information should be tailored based on demographic characteristics. This reflects the challenges designers of online comparative health care information are facing. Providing possibilities for information on demand and showing information about the medical specialist might be promising ways to increase the relevancy of online comparative health care information for patients. It is also important to examine in real life how different groups of people use comparative health care information in different health care choices.

## Abbreviations

NIVEL: Nederlands instituut voor onderzoek van de gezondheidszorg [Netherlands Institute for Health Services Research]
NPCF: Nederlandse Patiënten en Consumenten Federatie [Federation of Patients and Patient Organizations]
